# MET Overexpression Is Associated with Superior Immunotherapy Benefit in Advanced Non-Small Cell Lung Cancer

**DOI:** 10.3390/cancers17233801

**Published:** 2025-11-27

**Authors:** Hui Li, Lingzhi Hong, Pedro Rocha, Rafael Bach, Luisa M. Solis Soto, Waree Rinsurongkawong, Bingnan Zhang, Haniel A. Araujo, Yasir Y. Elamin, Mehmet Altan, Claudio A. Arrechedera, Jianling Zhou, Khaja B. Khan, Wei Lu, Elliana Young, Carl M. Gay, Tina Cascone, Lauren A. Byers, Ferdinandos Skoulidis, George Blumenschein, Frank V. Fossella, Anne Tsao, Marcelo V. Negrao, Natalie Vokes, Jia Wu, Hai T. Tran, Ignacio I. Wistuba, J. Jack Lee, Don L. Gibbons, Ara A. Vaporciyan, John V. Heymach, Xiuning Le, Jianjun Zhang

**Affiliations:** 1 Department of Thoracic/Head and Neck Medical Oncology, The University of Texas MD Anderson Cancer Center, Houston, TX 77030, USA; 2 Department of Imaging Physics, The University of Texas MD Anderson Cancer Center, Houston, TX 77030, USA; 3 Medical Oncology Department, Hospital del Mar, 08003 Barcelona, Spain; 4 IMIM (Instituto Hospital del Mar de Investigaciones Médicas), 08003 Barcelona, Spain; 5 Department of Translational Molecular Pathology, The University of Texas MD Anderson Cancer Center, Houston, TX 77030, USA; 6 Department of Systems Biology, The University of Texas MD Anderson Cancer Center, Houston, TX 77030, USA; 7 Department of Enterprise Data Engineering & Analytics, The University of Texas MD Anderson Cancer Center, Houston, TX 77030, USA; 8 Department of Genomic Medicine, The University of Texas MD Anderson Cancer Center, Houston, TX 77030, USA; 9 Department of Biostatistics, The University of Texas MD Anderson Cancer Center, Houston, TX 77030, USA; 10 Department of Molecular and Cellular Oncology, The University of Texas MD Anderson Cancer Center, Houston, TX 77030, USA; 11 Department of Thoracic and Cardiovascular Surgery, The University of Texas MD Anderson Cancer Center, Houston, TX 77030, USA

**Keywords:** MET overexpression, PD-L1, immune checkpoint inhibitors, non-small cell lung cancer

## Abstract

Background/Objectives: MET overexpression is common in non-small cell lung cancer (NSCLC). The correlation between MET overexpression and immune checkpoint inhibitor (ICI) efficacy in NSCLC is underexplored. Methods: In this retrospective observational cohort study, we curated a dataset of 279 stage IV NSCLC patients who received ICI treatment and had MET expression assessed by CLIA-certified immunohistochemistry (IHC) assay. MET expression was graded on a scale from 0 to 3+, with overexpression defined as 2+ or 3+. Clinicopathological features associated with MET expression were assessed using logistic regression. Overall survival (OS) and progression-free survival (PFS) were evaluated via Kaplan–Meier analysis and the multivariate Cox proportional hazards model. To derive the most parsimonious and statistically robust models, stepwise refinement based on the Akaike Information Criterion (AIC) was applied. Results: MET overexpression was observed in 220 of 279 patients (78.9%). Adenocarcinoma histology (*p* = 0.003) and PD-L1 expression (*p* < 0.001) were independently associated with MET overexpression. Kaplan–Meier analysis showed patients with MET overexpression had significantly superior OS (*p* = 0.012) and PFS (*p* = 0.033). Full Cox regression analysis revealed that MET overexpression was associated with longer OS (*p* = 0.040), independent of PD-L1 levels. Patients with MET overexpression had numerically longer PFS (*p* = 0.145). Following adjustment for metastatic burden, the AIC-selected OS and PFS models both demonstrated that MET overexpression remained a significant prognostic factor (OS: *p* = 0.010; PFS: *p* = 0.026). Conclusions: This real-world study suggests that MET overexpression, as assessed by IHC, is associated with better survival in advanced NSCLC patients treated with ICIs, independent of PD-L1 level. These results suggest the potential of MET expression as a predictive marker for ICI efficacy in advanced NSCLC patients and support the combination of MET-targeted agents with anti-PD1/PD-L1 ICIs as a promising strategy for NSCLC patients with MET overexpression.

## Simple Summary

MET is an oncogene frequently altered in NSCLC, but its relevance to immune checkpoint inhibitor (ICI) efficacy has not been systematically studied. In a cohort of 279 stage IV NSCLC patients treated with ICIs and evaluated for MET expression by CLIA-certified immunohistochemistry, we found that MET overexpression was associated with significantly improved overall and progression-free survival, contrasting with prior studies in localized or chemotherapy-treated disease. Although MET expression correlated with PD-L1 levels, it outperformed PD-L1 as a predictor of ICI benefit, and multivariate analysis confirmed MET as an independent prognostic factor. Patients with high expression of both MET and PD-L1 experienced the best survival outcomes, suggesting complementary biomarker utility. These findings identify MET overexpression as a promising predictive biomarker for ICI efficacy in advanced NSCLC and provide strong rationale for investigating MET-targeted strategies, including antibody–drug conjugates, in combination with ICIs.

## 1. Introduction

Lung cancer is the leading cause of cancer-related mortality [1]. Most of the NSCLC patients are diagnosed at an advanced stage. Non-small cell lung cancer (NSCLC) accounts for 85% of lung cancer diagnoses [2]. With the advancement of treatment, including targeted therapy and immunotherapy, the overall survival of NSCLC patients improved substantially in recent years [3]. For the driver-negative NSCLC, the immune checkpoint inhibitors (ICIs), which include anti-programmed death-1 (anti-PD-1), anti-programmed death-ligand 1 (anti-PD-L1), and anti-cytotoxic T-lymphocyte associated protein 4 (anti-CTLA-4) agents have become the treatment cornerstones [4]. However, only a small subset of patients can achieve durable clinical benefit. High PD-L1 expression and high tumor mutation burden are associated with increased benefit from ICI treatment [5]. On the other hand, the presence of many oncodriver mutations is associated with less benefit from ICI [6,7].

MET is a proto-oncogene that is expressed by various cells [8]. Various biological alterations in MET have been discovered, including exon 14 skipping mutations, activating mutations in the kinase domain, gene amplification, fusions, and protein overexpression. MET alterations, including MET exon 14 skipping and amplifications, have been reported to be associated with poor prognosis in NSCLC patients [9,10]. MET exon 14 skipping mutations and MET amplification have emerged as critical therapeutic targets in NSCLC, with the development of MET tyrosine kinase inhibitors (TKIs), monoclonal antibodies, and antibody–drug conjugates (ADCs) [11].

Beyond being a therapeutic target, MET alterations have also been reported to be associated with acquired resistance to several targeted therapies such as EGFR inhibitors, KRAS inhibitors and ALK inhibitors [12]. In addition, dysregulated MET signaling has been found to contribute to chemotherapy resistance [13]. On the other hand, the influence of MET alterations on the efficacy of ICIs are not well understood. While MET alterations encompass a broad spectrum, MET protein overexpression is the most prevalent MET abnormality observed in advanced NSCLC and is easily assessed in clinical practice through immunohistochemistry (IHC).

Importantly, MET overexpression has been implicated in tumor progression and immune evasion [8], yet its clinical relevance as a predictive biomarker for ICI response remains uncertain. Given its high prevalence, assay accessibility, and strong biological rationale, we aimed to specifically investigate the role of MET overexpression in this context. In the current study, we assessed whether MET overexpression, assessed by CLIA-certified IHC assay, was associated with the clinical efficacy of ICIs in advanced NSCLC patients.

## 2. Materials and Methods

### 2.1. Study Cohort

This retrospective cohort study was conducted at MD Anderson Cancer Center and approved by the institution’s Ethics Committees. Written informed consent was obtained from all patients. The inclusion criteria are as follows: 1. The patients were histologically diagnosed with NSCLC according to WHO classification. 2. All patients were diagnosed with stage IV lung cancer. 3. Patients received either ICI monotherapy or ICIs administered in combination with chemotherapy. 4. All the tissue samples, including the core-needle biopsies and surgical resections were tested with MET expression by IHC. To minimize selection bias, all consecutive patients who met the eligibility criteria between October 2014 and September 2023 at MD Anderson Cancer Center were included. A total of 279 patients were enrolled and received ICI treatment during this period.

The clinical data, including the age, gender, smoking history, metastatic (M) stage, PD-L1 expression, brain metastases and others evaluated as potential predictors were extracted from electronic medical records. Histologic subtypes were obtained from the pathologic reports. Follow-up information and survival data were also collected. The patients were followed up until November 2025. The primary endpoints included progression-free survival (PFS), and overall survival (OS). For the evaluation of outcomes, PFS was defined as the time from ICI initiation to disease progression, death from any cause, or the most recent imaging without progression. OS was defined as the time from ICI initiation to death or the last follow-up date.

### 2.2. MET IHC Expression and Gene Alterations

MET expression from tissue specimens was assessed by CLIA-certified IHC assay by experienced thoracic pathologists at MD Anderson Cancer Center. The scoring was analyzed by a qualitative method based on the immunoreactivity observed in the specimen according to MetMab IHC defined scoring criteria with a scale of 0 to 3+. This method was used to assess both the proportion of tumor cells stained and the intensity of the staining. The categories were defined as follows: 0 meant no staining or less than 50% of tumor cells showing with membrane and/or cytoplasmic staining (could be combination of any staining intensities); 1+ indicated 50% or more of tumor cells with membrane and/or cytoplasmic staining with weak or higher intensity but <50% tumor cells with moderate or higher intensity; 2+ meant 50% or more of tumor cells with membrane and/or cytoplasmic staining with moderate or higher intensity but <50% tumor cells with strong intensity; 3+ represented 50% or more of tumor cells with membrane and/or cytoplasmic staining with strong intensity [14]. Consequently, samples showing 2+ or 3+ staining were classified as MET high expression or overexpression. MET gene alterations, including amplifications, exon 14 skipping, and other targetable genomic alterations were collected from MD Anderson Molecular Diagnostic Laboratory gene panel or Guardant 360 panel. MET amplification and other genomic fusions were also assessed using fluorescence in situ hybridization (FISH). MET amplification was defined as having a MET copy number of ≥5 and/or a MET signal ratio of ≥2.

### 2.3. Statistical Analysis

Continuous variables were summarized using means with standard deviations or medians with interquartile ranges, depending on the results of normality testing. Categorical variables were reported as frequencies and percentages. To assess the associations between MET overexpression and clinicopathological factors, appropriate statistical tests were employed, including Student’s *t*-test or the Mann–Whitney U test for continuous variables, and the chi-square or Fisher’s exact test for categorical variables. Multivariate logistic regression models were also used, adjusting for potential confounders such as PD-L1 expression, histologic subtype, M stage and smoking status. Results were reported as odds ratios (ORs) with 95% confidence intervals (CIs).

To analyze survival outcomes, including OS and PFS, Kaplan–Meier survival curves were generated and compared using the log-rank test. Patients without confirmed events were censored at the date of last follow-up. To evaluate the independent prognostic value of clinical variables, we first constructed full multivariable Cox proportional hazards models for OS and PFS. Among all the predictors, PD-L1 expression was the only covariate that showed imbalance between low and high MET expression groups. To address potential confounding, PD-L1 was included as an adjustment variable in the multivariate models. Matching was not applied, as other covariates were well balanced and multivariable regression adequately controlled for PD-L1-related bias. The following covariates were included in the full Cox models: age, sex, smoking history, histologic subtype, M stage at ICI initiation, PD-L1 expression, treatment regimen, treatment line, presence of brain metastases, and MET IHC expression. Hazard ratios (HRs), 95% confidence intervals (CIs), and *p*-values were reported.

To derive the most parsimonious and statistically robust models while preserving explanatory value, stepwise variable selection using the Akaike Information Criterion (AIC) was conducted. The models with the lowest AIC were selected as the final OS and PFS models and used for interpretation of prognostic factors. The AIC-selected OS model retained age, M stage, and MET expression, while the AIC-selected PFS model retained M stage and MET expression. The proportional hazards (PH) assumption was evaluated using scaled Schoenfeld residuals for each covariate in the AIC-selected models. All statistical analyses were performed using R software (version 4.2.1, R Foundation for Statistical Computing, Vienna, Austria) and two-sided *p*-values < 0.05 were considered statistically significant.

## 3. Results

### 3.1. Clinicopathological Features of Recruited Patients

We identified 279 patients with stage IV NSCLC, who received ICIs between October 2014 and September 2023 at MD Anderson. The median follow-up time was 764 days. Adenocarcinoma constituted the majority of cases, occurring in 216 patients (77.4%), while squamous cell carcinoma was present in 43 patients (15.4%). Overall, 148 patients (53.0%) were male, and 136 (48.7%) were aged ≥65 years. At the initiation of immunotherapy, 73 patients (26.2%) had brain metastases, and 130 (46.6%) presented with M1c disease. The clinical characteristics of these patients are listed in Table 1. Detailed immune checkpoint inhibitor treatment regimens for the cohort are presented in Supplementary Table S1.

### 3.2. MET Overexpression Is Associated with Higher PD-L1 Level

**Figure 1:**
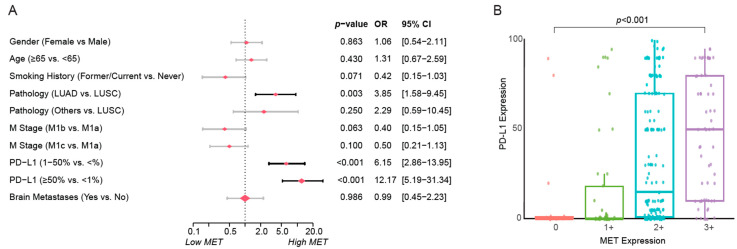
Associations between MET IHC expression and clinicopathological variables in patients receiving ICIs. (**A**) The logistic regression analysis and the forest plot illustrated the relationship between various variables and MET IHC expression. The plot displayed the effect estimates as the Odds Ratios (ORs). Error bars represent the 95% CIs, indicating the precision of the OR estimates. *p*-values were calculated to determine the statistical significance of each predictor’s association with the high/low MET expression. The size of the diamonds is proportional to the effect size. (**B**) The box plot illustrates the correlation between PD-L1 and MET expression, indicating that lung cancers with high MET expression are more likely to exhibit high PD-L1 expression. LUAD: lung adenocarcinoma, LUSC: lung squamous cell carcinoma.

A total of 220 (78.9%) patients exhibited MET overexpression (2+ or 3+ by IHC), while 59 (21.1%) patients showed low MET IHC expression (0 or 1+). Tumors with high PD-L1 expression (TPS ≥ 50%) had a higher prevalence of MET overexpression (43.6% vs. 16.9%, *p* < 0.001) (Table 1). Multivariate analysis revealed significant correlations of MET overexpression with adenocarcinoma histology (OR: 3.85, 95% CI: 1.58–9.45, *p* = 0.003), and with high PD-L1 expression (≥50%, OR:12.17, 95% CI: 5.19–31.34, *p* < 0.001) (Figure 1A). Using PD-L1 expression as a continuous variable, further analysis revealed that MET overexpression was associated with high level of PD-L1 expression. (*p* < 0.001) (Figure 1B).

### 3.3. MET Overexpression Does Not Correlate with MET Gene Alterations

Next, we assessed the correlation between MET expression and MET gene alterations. MET alterations were detected in 23 (8.2%) patients in this cohort. Among the cases with MET overexpression, 7 (3.2%) patients were detected with MET exon 14 skipping mutations and 6 (2.7%) patients with MET amplification. On the other hand, no patients in the MET low expression group had MET exon 14 skipping mutations or MET amplification. MET exon 14 skipping mutation or amplification was not associated with MET protein overexpression (Supplementary Table S2). Aside from the above MET gene alterations, the relationships between other targetable genomic alterations and MET IHC expression are summarized in Supplementary Table S3.

### 3.4. MET Overexpression Is Associated with Longer Survival from ICI Treatment Independent of PD-L1 Levels

**Figure 2:**
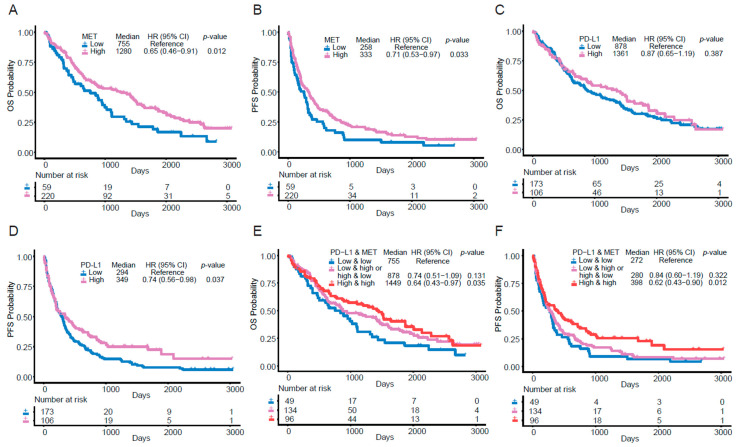
Kaplan–Meier analysis of factors correlated with OS and PFS in patients receiving ICIs. (**A**) OS and (**B**) PFS were stratified by MET expression (High MET expression: 2+ or 3+). (**C**) OS and (**D**) PFS were stratified by PD-L1 expression (High PD-L1 expression: ≥50%). (**E**) OS and (**F**) PFS were stratified by three different groups: low PD-L1& low MET expression, low PD-L1/high MET or low MET/high PD-L1 expression, and high PD-L1 & high MET expression (High MET expression:2+ or 3+; High PD-L1 expression: ≥50%). Univariate Cox proportional hazards regression models were also employed to analyze the hazard ratio (HR), 95% CI and *p*-values.

Patients with MET overexpression had significantly longer OS (Median OS: 1280 vs. 755 days, *p* = 0.012) and longer PFS (Median PFS: 333 vs. 258 days, *p* = 0.033) (Figure 2A,B). On the other hand, high PD-L1 (≥50%) was correlated with longer PFS (Median PFS: 349 vs. 294 days, *p* = 0.037) and numerically longer OS (*p* = 0.387) (Figure 2C,D). Furthermore, tumors with both MET overexpression (2+ or 3+) and high PD-L1 expression (≥50%) had the longest OS (Median OS: 1449 vs. 755 days, *p* = 0.035) and PFS (Median PFS: 398 vs. 272 days, *p* = 0.012) (Figure 2E,F). Additional subgroup analysis is presented in Supplementary Figure S1.

**Figure 3:**
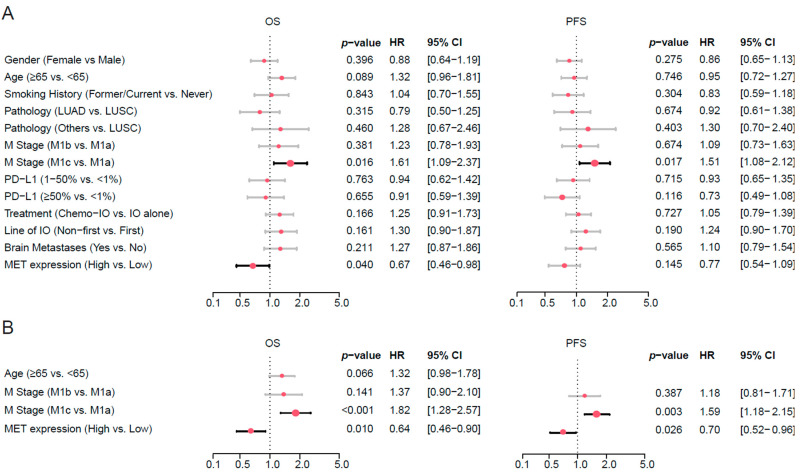
Multivariate Cox regression analysis of factors correlated with OS and PFS in patients receiving ICIs. (**A**) The forest plot showed all covariates included in the full Cox models. Positive associations (HR > 1) indicate increased risk, whereas HR < 1 indicates reduced risk. M1c disease and high MET expression were significantly associated with OS, and similar trends were observed for PFS. (**B**) The panels display the variables included in each AIC-selected model. M1c metastatic disease consistently predicted worse OS and PFS, while high MET expression was associated with favorable outcomes across both endpoints. The plots display the effect estimates as HRs, with error bars representing the 95% CIs to indicate the precision of the HR estimates. *p*-values were calculated to determine the statistical significance of each predictor’s association with OS or PFS. The size of the dots is proportional to the effect size. LUAD: lung adenocarcinoma, LUSC: lung squamous cell carcinoma, ICI: Immune checkpoint inhibitor, ICI-Chemo: Immune checkpoint inhibitor combined with chemotherapy.

Furthermore, multivariate analysis using full Cox model revealed that only M1c stage and high MET expression reached statistical significance for predicting OS. MET overexpression was associated with better OS (HR: 0.67, 95% CI: 0.46–0.98, *p* = 0.040), independent of PD-L1 expression (Figure 3A). Other variables including PD-L1 expression, brain metastases, treatment regimen, and smoking status did not reach statistical significance. Regarding PFS, only M1c stage reached statistical significance. MET overexpression showed a trend toward improved PFS (HR: 0.77, 95% CI: 0.54–1.09, *p* = 0.145) (Figure 3A).

To derive the most parsimonious model while retaining covariates that meaningfully contributed to model fit, we performed stepwise variable selection using AIC. Models with the lowest AIC were selected as the final OS and PFS models. The proportional hazards (PH) assumption was evaluated using scaled Schoenfeld residuals for each covariate and the PH plots are reported in Supplementary Figure S2. The AIC-selected OS model identified three independent predictors of OS: Age, M stage and MET expression. This simplified model demonstrated MET overexpression remained an independent favorable prognostic factor (HR: 0.64, 95% CI: 0.46–0.90, *p* = 0.010) after adjustment for metastatic burden. The AIC-selected PFS model identified two independent predictors of PFS: M stage and MET expression. These results were consistent with OS, showing MET overexpression remained an independent favorable prognostic factor (HR: 0.70, 95% CI: 0.52–0.96, *p* = 0.026) after adjustment for metastatic burden. The AIC-selected PFS model consistently supported MET overexpression as a significant prognostic marker among patients treated with ICIs (Figure 3B).

## 4. Discussion

The incidence of MET overexpression and its clinical impact in NSCLC vary widely across different studies [15,16,17,18,19,20,21,22,23]. In localized NSCLC, MET overexpression is not significantly associated with OS or indicative of worse OS [19,21]. In a cohort of 791 newly diagnosed, treatment-naive NSCLC patients, of whom 24.2% were at stage IV, Song et al. found that positive MET expression was associated with poorer OS. Multivariate analysis identified MET expression as an independent prognostic factor for reduced [22]. Similarly, other studies including cohorts across all disease stages have also reported that high MET expression is associated with worse OS [21,23]. However, there are very few studies specifically focusing on stage IV NSCLC patients, particularly those treated with ICIs.

Our Kaplan–Meier analyses demonstrated that patients with MET overexpression experienced significantly improved OS and PFS with immunotherapy. To further investigate whether the survival advantage associated with MET overexpression was independent of other known prognostic variables, we conducted multivariable Cox proportional hazards modeling. The simplified AIC selected models, which retained only covariates that significantly contributed to model fit, consistently identified MET overexpression as an independent favorable prognostic factor for both OS and PFS. Importantly, this association persisted even after adjusting for metastatic disease burden, a well-recognized determinant of survival in advanced NSCLC. In line with our findings, Reis et al. studied a small cohort of NSCLC treated with ICI (*N* = 51) and observed a numerically improved OS in patients with MET overexpression. And no association was observed between MET expression and outcomes in patients receiving chemotherapy [24]. Since MET overexpression has either no impact on survival or is associated with inferior survival in NSCLC following surgery or chemotherapy [19,20,21,22,23,24], the improved survival is unique to ICI-treated patients.

We also found that MET exon 14 skipping mutations, MET amplification, and other targetable alterations were not associated with MET protein overexpression, which is consistent with prior studies [25,26], although the sample size was relatively small across all cohorts. Therefore, MET overexpression should not be considered a surrogate marker for MET-driven oncogenic events or for other targetable alterations. In addition, patients with MET exon 14 skipping mutations or high-level MET amplification typically have poor clinical outcomes, consistent with their known aggressive disease biology [27,28]. In contrast, MET overexpression in our study identified a subgroup of patients with more favorable responses to immunotherapy. Taken together, these findings suggest that MET protein overexpression represents a biologically distinct phenotype rather than a proxy for METex14 or MET amplification. This distinction is important for understanding the prognostic and predictive roles of MET alterations and may help refine the clinical interpretation of MET dysregulation in advanced NSCLC.

The biological basis underlying the result that MET overexpressing tumors experience improved survival with ICI treatment remains unclear and warrants further investigation. MET alterations have been reported to be associated with enhanced tumor immunogenicity, enriched infiltration of immune cells, and improved immune responses [29]. In our study, MET overexpression was associated with high level of PD-L1, an important predictive marker for ICI treatment. The underlying mechanisms between MET expression and PD-L1 expression in lung cancer remain poorly understood. Martin et al. observed that PD-L1 expression was upregulated in MET-amplified lung carcinoma cells upon IFNγ treatment, with this induction impaired by MET inhibition, suggesting an interaction between MET signaling and the JAK/STAT3 pathway downstream of IFNγ, leading to increased PD-L1 expression in tumors [30]. In contrast, Saigi et al. found that MET activation in MET-altered lung cancer cells upregulated PD-L1 expression independently of the IFNγ-mediated JAK/STAT pathway [31]. Ahn et al. demonstrated that MET activation via HGF consistently increased PD-L1 expression in lung adenocarcinoma cell lines, implicating a role of MET overexpression or activation in immune escape through PD-L1 upregulation [32]. Similarly, Peng et al. also reported that MET amplification in NSCLC upregulated PD-L1 expression and promoted immune escape [33]. Better understanding the mechanisms underlying MET-driven immune response and immune escape could aid in developing more effective immunotherapies.

Importantly, in this study, MET overexpression showed stronger associations with clinical outcomes than PD-L1 within this cohort. Moreover, the association between MET overexpression and improved survival was independent of PD-L1, suggesting that MET overexpression may influence the tumor immune microenvironment through mechanisms beyond the PD-L1 pathway. These findings not only highlight the potential of MET expression as a predictive biomarker beyond PD-L1 but also suggest a possible synergistic effect between anti-PD-1/PD-L1 therapies and MET-targeting agents. Multiple therapeutic agents targeting MET are under investigation, including MET TKIs, monoclonal antibodies and ADCs [34]. Although MET overexpression as a biomarker for MET monoclonal antibodies or MET-TKIs has not been successfully established [35], MET overexpression has been reported to be associated with clinical outcome from MET ADCs. For instance, in the Phase II LUMINOSITY trial, a MET ADC Teliso-V has demonstrated durable responses in NSCLC patients, particularly those with MET overexpression [36]. Our results therefore support the combination of MET-targeting agents, particularly ADCs with anti-PD1/PD-L1 ICIs in NSCLC with MET overexpression. The most utilized standard of care for advanced stage NSCLC is anti-PD-1/PD-L1 plus chemotherapy [37]. Our results suggest that in patients with MET overexpression, chemotherapy may be potentially replaced by MET-ADCs with less systemic toxicity as well as synergistic effect with anti-PD1/PD-L1 ICIs.

Our study has several limitations that should be acknowledged. While we identified that MET IHC expression may serve as a predictive marker for the efficacy of ICIs in lung cancer, the underlying mechanisms and its correlation with PD-L1 expression have not been thoroughly investigated and warrant further research. In addition, this was a retrospective, single-center study, which limits the generalizability of the findings. Although our analyses adjusted for known confounders, potential bias from unmeasured confounding remains a possibility. Future studies should aim to validate these findings in larger, independent multi-center cohorts and explore potential synergistic effects of MET and PD-L1 co-targeting in prospective clinical trials before informing clinical practice.

## 5. Conclusions

This study, to our knowledge, represents the largest cohort to date examining the association between MET protein expression and ICI efficacy in advanced NSCLC patients. We demonstrated that MET overexpression was associated with improved OS and PFS in NSCLC patients treated with ICIs, with predictive performance surpassing that of PD-L1. Importantly, although MET overexpression correlated with high PD-L1 levels, its association with ICI efficacy was independent of PD-L1. Patients with both MET overexpression and high PD-L1 exhibited the best survival outcomes after ICI treatment, suggesting an independent and synergistic role of MET overexpression beyond the PD-1/PD-L1 pathway. These findings support the strategy of combining PD-1/PD-L1 ICIs with MET-targeting agents, particularly MET ADCs, for patients with MET overexpression.

## Figures and Tables

**Table 1:** The demographics and clinicopathological variables and univariate analysis of the variables correlated with the MET IHC expression.

	Overall (*N* = 279)	Low MET Expression (*N* = 59)	High MET Expression (*N* = 220)	*p*-Value
Gender				
Female	131 (47.0%)	25 (42.4%)	106 (48.2%)	0.518
Male	148 (53.0%)	34 (57.6%)	114 (51.8%)	
Age				
<65	143 (51.3%)	30 (50.8%)	113 (51.4%)	1.000
≥65	136 (48.7%)	29 (49.2%)	107 (48.6%)	
Smoking history				
Never	61 (21.9%)	7 (11.9%)	54 (24.5%)	0.056
Former & Current	218 (78.1%)	52 (88.1%)	166 (75.5%)	
Histology				
LUAD	216 (77.4%)	42 (71.2%)	174 (79.1%)	0.286
LUSC	43 (15.4%)	13 (22.0%)	30 (13.6%)	
Others	20 (7.2%)	4 (6.8%)	16 (7.3%)	
M stage				
M1a	94 (33.7%)	16 (27.1%)	78 (35.5%)	0.331
M1b	55 (19.7%)	15 (25.4%)	40 (18.2%)	
M1c	130 (46.6%)	28 (47.5%)	102 (46.4%)	
PD-L1 expression				
<1%	72 (25.8%)	33 (55.9%)	39 (17.7%)	<0.001
1–50%	101 (36.2%)	16 (27.1%)	85 (38.6%)	
≥50%	106 (38.0%)	10 (16.9%)	96 (43.6%)	
Treatment plan				
ICI alone	135 (48.4%)	28 (47.5%)	107 (48.6%)	0.989
ICI-Chemo	144 (51.6%)	31 (52.5%)	113 (51.4%)	
Line of treatment				
First line	213 (76.3%)	47 (79.7%)	166 (75.5%)	0.615
Non-first line	66 (23.7%)	12 (20.3%)	54 (24.5%)	
Brain metastases				
Yes	73 (26.2%)	16 (27.1%)	57 (25.9%)	0.983
No	206 (73.8%)	43 (72.9%)	163 (74.1%)	

LUAD: lung adenocarcinoma, LUSC: lung squamous cell carcinoma, ICI: Immune checkpoint inhibitor, ICI-Chemo: Immune checkpoint inhibitor combined with chemotherapy.

## Data Availability

The data may be available from the corresponding author based on reasonable request.

## Supplementary Materials

The following supporting information can be downloaded at: https://www.mdpi.com/article/10.3390/cancers17233801/s1, Supplementary Table S1. Summary of ICI treatment regimens in the cohort. Supplementary Table S2. The univariate analysis of the MET gene alterations and the MET IHC expression. Supplementary Table S3. The univariate analysis of targetable genomic alterations in relation to MET IHC expression. Supplementary Figure S1. Survival analysis of OS and PFS stratified by MET IHC expression in different subgroups. Supplementary Figure S2. Proportional hazards (PH) diagnostic plots for the AIC-selected OS and PFS Cox models.
