# Supplementary material for: MET Overexpression Is Associated with Superior Immunotherapy Benefit in Advanced Non-Small Cell Lung Cancer
Source: Cancers (Basel). 2025 Nov 27;17(23):3801. doi: 10.3390/cancers17233801 (PMC12691313; doi:10.3390/cancers17233801)
Supplement: Supplementary file 1 [file cancers-17-03801-s001.zip › Supplementary Data.pdf]

Supplementary Data:

Supplementary Table S1. Summary of ICI treatment regimens in the cohort.

Immunotherapy Regimen	Count	Percent (%)
Pembrolizumab + chemotherapy	135	48.4
Pembrolizumab	65	23.3
Ipilimumab + nivolumab	31	11.1
Nivolumab	30	10.8
Atezolizumab	9	3.2
Atezolizumab + chemotherapy	7	2.5
Durvalumab + tremelimumab	1	0.4
Nivolumab + chemotherapy	1	0.4

Supplementary Table S2. The univariate analysis of the MET gene alterations and the MET IHC expression.

	Overall (*N*=279)	Low MET expression (*N*=59)	High MET expression (*N*=220)	*P*-value
Exon 14 skipping				
No	264 (94.6%)	55 (93.2%)	209 (95.0%)	0.351
Yes	7 (2.5%)	0 (0%)	7 (3.2%)	
Missing	8 (2.9%)	4 (6.8%)	4 (1.8%)	
Amplification				
No	265 (95.0%)	55 (93.2%)	210 (95.5%)	0.605
Yes	6 (2.2%)	0 (0%)	6 (2.7%)	
Missing	8 (2.9%)	4 (6.8%)	4 (1.8%)	

Supplementary Table S3. The univariate analysis of targetable genomic alterations in relation to MET IHC expression

	Overall (*N*=279)	Low MET expression (*N*=59)	High MET expression (*N*=220)	*P*-value
KRAS G12C mutation				
No	250 (89.6%)	51 (86.4%)	199 (90.5%)	0.6

	Overall (*N*=279)	Low MET expression (*N*=59)	High MET expression (*N*=220)	*P*-value
Yes	26 (9.3%)	7 (11.9%)	19 (8.6%)	
Missing	3 (1.1%)	1 (1.7%)	2 (0.9%)	
EGFR mutations				
No	260 (93.2%)	56 (94.9%)	204 (92.7%)	0.536
Yes	16 (5.7%)	2 (3.4%)	14 (6.4%)	
Missing	3 (1.1%)	1 (1.7%)	2 (0.9%)	
BRAF V600E mutation				
No	269 (96.4%)	57 (96.6%)	212 (96.4%)	1
Yes	7 (2.5%)	1 (1.7%)	6 (2.7%)	
Missing	3 (1.1%)	1 (1.7%)	2 (0.9%)	
ERBB2 exon 20 insertion				
No	269 (96.4%)	55 (93.2%)	214 (97.3%)	0.164
Yes	7 (2.5%)	3 (5.1%)	4 (1.8%)	
Missing	3 (1.1%)	1 (1.7%)	2 (0.9%)	
ALK fusions				
No	275 (98.6%)	58 (98.3%)	217 (98.6%)	1
Yes	1 (0.4%)	0 (0%)	1 (0.5%)	
Missing	3 (1.1%)	1 (1.7%)	2 (0.9%)	

Supplementary Figure S1. Survival analysis of OS and PFS stratified by MET IHC expression in different subgroups. In the Kaplan-Meier plots, **(A)** OS and **(B)** PFS were stratified by the high MET and low MET expression in NSCLC male patients; **(C)** OS and **(D)** PFS were stratified by the high MET and low MET expression in NSCLC patients with former & current smoking history; **(E)** OS and **(F)** PFS were stratified by the high MET and low MET expression in lung adenocarcinoma patients. Univariate Cox proportional hazards regression models were employed to analyze the hazard ratio (HR), 95% CIs and *P*-values.

Supplementary Figure S2. Proportional hazards (PH) diagnostic plots for the AIC-selected OS and PFS Cox models. Scaled Schoenfeld residuals plotted against event time are shown for the three covariates retained in the final AIC-selected OS model and two covariates retained in the final AIC-selected PFS model. Points represent scaled residuals for individual events, while the solid blue line depicts a non-parametric LOESS smoother with 95% confidence bands (shaded area). The horizontal dashed line represents the expected value of zero under the proportional hazards assumption. A roughly horizontal trend suggests no meaningful time-dependent deviation from proportionality. Together, these diagnostics support that the proportional hazards assumption is adequately met for the final OS and PFS model.
