# Supplementary figures and images for: MET Overexpression Is Associated with Superior Immunotherapy Benefit in Advanced Non-Small Cell Lung Cancer
Source: Cancers (Basel). 2025 Nov 27;17(23):3801. doi: 10.3390/cancers17233801 (PMC12691313; doi:10.3390/cancers17233801)

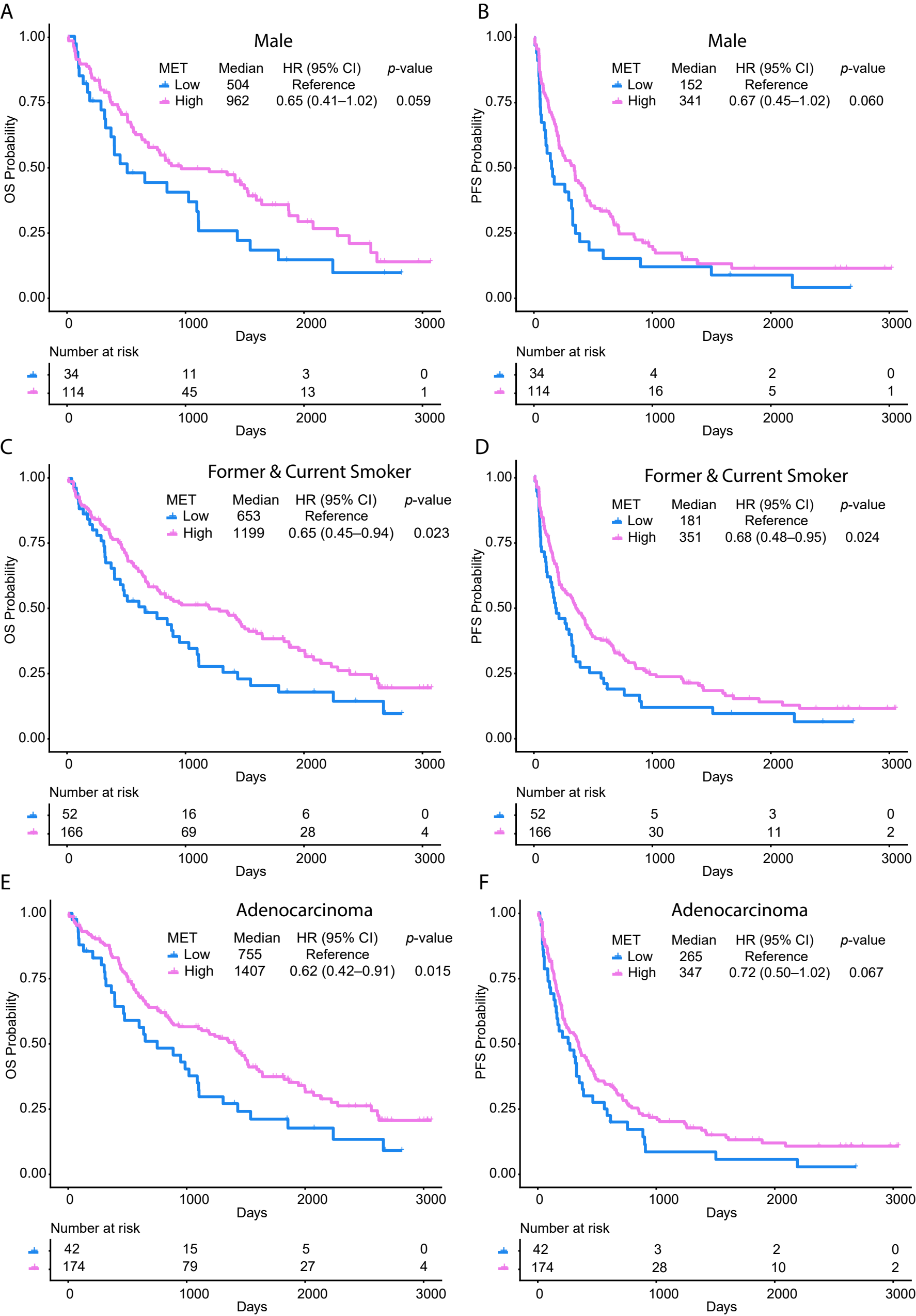

Supplement: Supplementary file 1 [file cancers-17-03801-s001.zip › MET Supplementary Figure S1.pdf]

**OS: Age**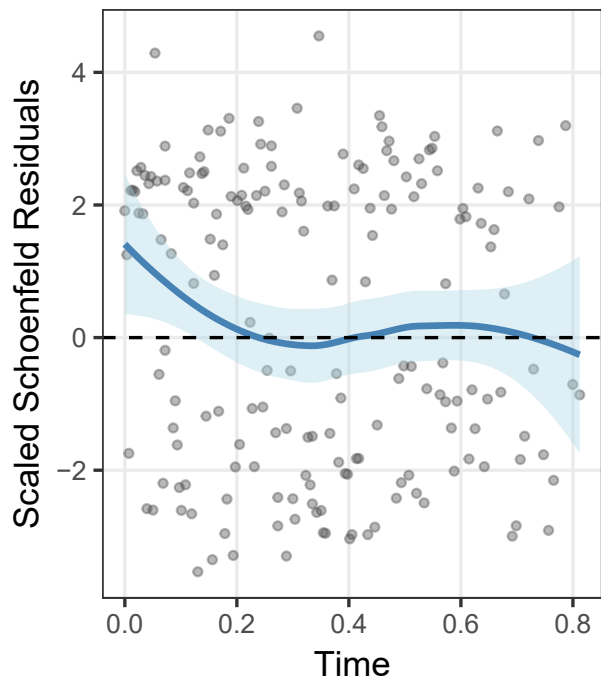**OS: M stage**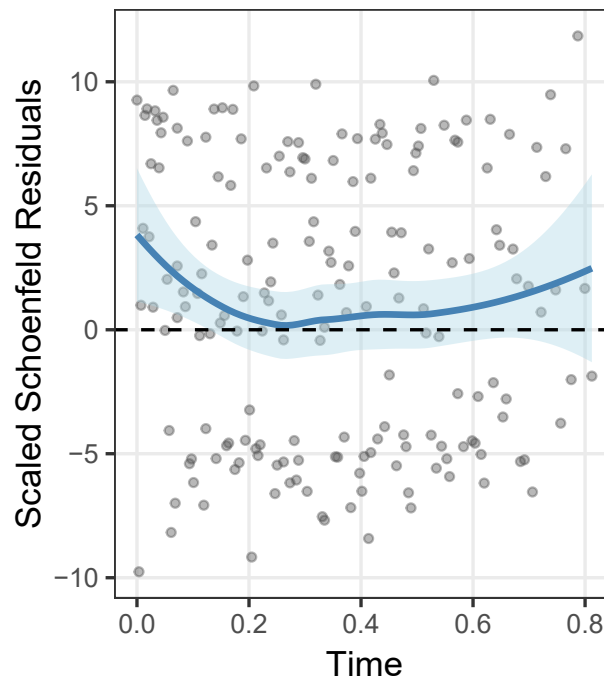**OS: MET expression**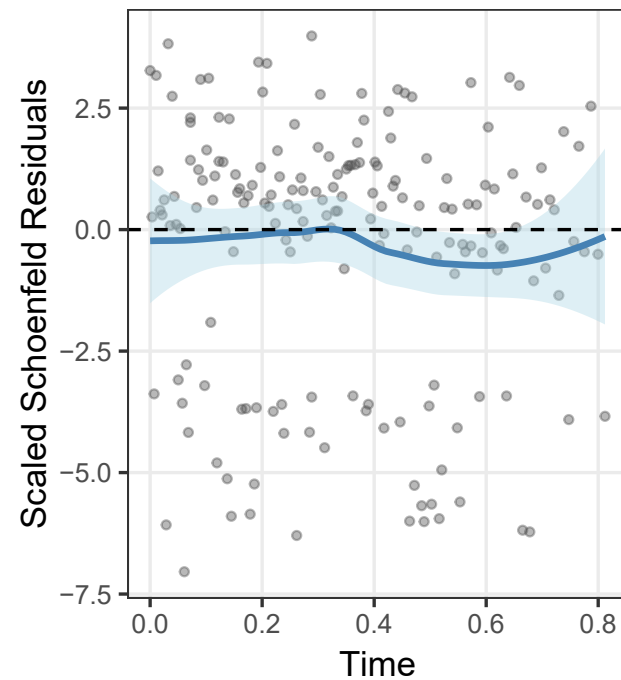**PFS: M stage**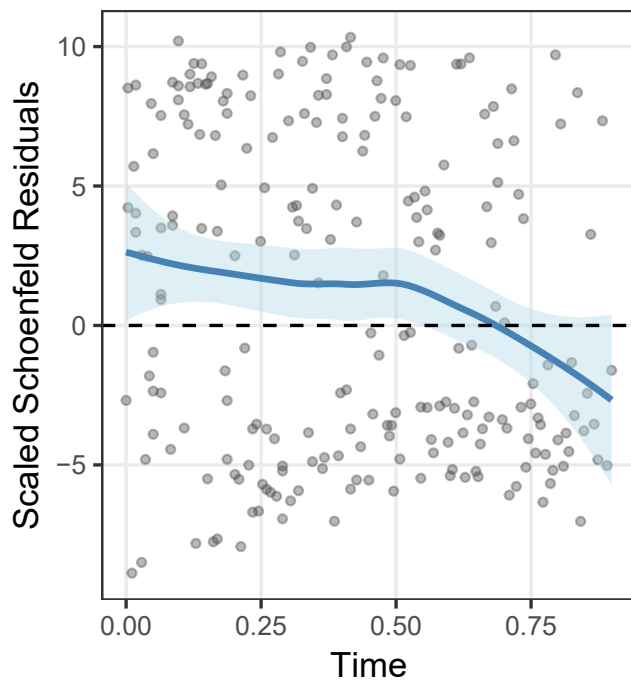**PFS: MET expression**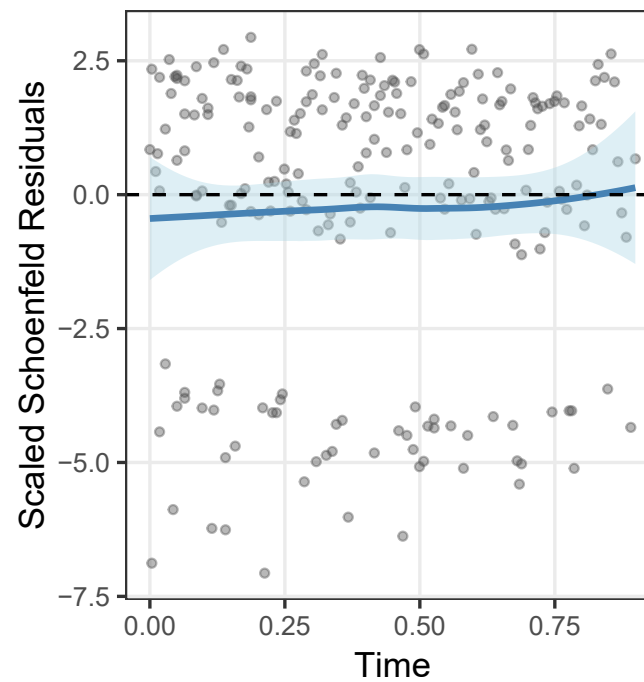

Supplement: Supplementary file 1 [file cancers-17-03801-s001.zip › MET Supplementary Figure S2.pdf]
